# Compliance With Mobile Ecological Momentary Assessment of Self-Reported Health-Related Behaviors and Psychological Constructs in Adults: Systematic Review and Meta-analysis

**DOI:** 10.2196/17023

**Published:** 2021-03-03

**Authors:** Marie T Williams, Hayley Lewthwaite, François Fraysse, Alexandra Gajewska, Jordan Ignatavicius, Katia Ferrar

**Affiliations:** 1 Innovation, Implementation And Clinical Translation in Health Allied Health and Human Performance University of South Australia Adelaide Australia; 2 Department of Kinesiology and Physical Education Faculty of Education McGill University Montreal, QC Canada; 3 Alliance for Research in Exercise, Nutrition and Activity Allied Health and Human Performance University of South Australia Adelaide Australia

**Keywords:** mobile momentary ecological assessment, adult, compliance, systematic review, meta-analysis, mobile phone

## Abstract

**Background:**

Mobile ecological momentary assessment (mEMA) permits real-time capture of self-reported participant behaviors and perceptual experiences. Reporting of mEMA protocols and compliance has been identified as problematic within systematic reviews of children, youth, and specific clinical populations of adults.

**Objective:**

This study aimed to describe the use of mEMA for self-reported behaviors and psychological constructs, mEMA protocol and compliance reporting, and associations between key components of mEMA protocols and compliance in studies of nonclinical and clinical samples of adults.

**Methods:**

In total, 9 electronic databases were searched (2006-2016) for observational studies reporting compliance to mEMA for health-related data from adults (>18 years) in nonclinical and clinical settings. Screening and data extraction were undertaken by independent reviewers, with discrepancies resolved by consensus. Narrative synthesis described participants, mEMA target, protocol, and compliance. Random effects meta-analysis explored factors associated with cohort compliance (monitoring duration, daily prompt frequency or schedule, device type, training, incentives, and burden score). Random effects analysis of variance (*P*≤.05) assessed differences between nonclinical and clinical data sets.

**Results:**

Of the 168 eligible studies, 97/105 (57.7%) reported compliance in unique data sets (nonclinical=64/105 [61%], clinical=41/105 [39%]). The most common self-reported mEMA target was affect (primary target: 31/105, 29.5% data sets; secondary target: 50/105, 47.6% data sets). The median duration of the mEMA protocol was 7 days (nonclinical=7, clinical=12). Most protocols used a single time-based (random or interval) prompt type (69/105, 65.7%); median prompt frequency was 5 per day. The median number of items per prompt was similar for nonclinical (8) and clinical data sets (10). More than half of the data sets reported mEMA training (84/105, 80%) and provision of participant incentives (66/105, 62.9%). Less than half of the data sets reported number of prompts delivered (22/105, 21%), answered (43/105, 41%), criterion for valid mEMA data (37/105, 35.2%), or response latency (38/105, 36.2%). Meta-analysis (nonclinical=41, clinical=27) estimated an overall compliance of 81.9% (95% CI 79.1-84.4), with no significant difference between nonclinical and clinical data sets or estimates before or after data exclusions. Compliance was associated with prompts per day and items per prompt for nonclinical data sets. Although widespread heterogeneity existed across analysis (I^2^>90%), no compelling relationship was identified between key features of mEMA protocols representing burden and mEMA compliance.

**Conclusions:**

In this 10-year sample of studies using the mEMA of self-reported health-related behaviors and psychological constructs in adult nonclinical and clinical populations, mEMA was applied across contexts and health conditions and to collect a range of health-related data. There was inconsistent reporting of compliance and key features within protocols, which limited the ability to confidently identify components of mEMA schedules likely to have a specific impact on compliance.

## Introduction

### Background

Ecological momentary assessment (EMA) is a survey method that allows collection of data on participant behaviors, affect, and perceptual experiences in real-time (momentary) and real-life environments (ecological) [1]. In its original form, EMA required pen and paper diaries or logs to be completed on random (signal) or fixed (interval) time-based schedules or in response to a specific target behavior, psychological or social event (event-based). With the advent of handheld technologies, mobile EMA (mEMA) and increasingly mobile ecological momentary interventions (mEMIs) can be completed through automated schedules via handheld devices such as tablets and mobile phones.

As mEMA or mEMI have the potential to capture data in real time, the level of recall bias is potentially reduced. In addition, contextual (where and who the respondent is with) and antecedents to the specific target behavior or psychological construct can be obtained [1,2]. As a survey approach, mEMA or mEMI has undeniable utility, but data are dependent on participants consistently responding to the mEMA or mEMI schedule (compliance) [3]. Although electronically delivered surveys to personal mobile devices provide a means of time or date stamping and limit the possibility of hoarding, back and forward filling [4], concerns have been raised about protocol burden, missing data (especially if systematic), mindless answering, and survey habituation when lengthier questionnaires can be circumvented by a no response to initial questions [2]. EMA data with low compliance rates are unlikely to be ecologically valid; however, it is also possible to have good individual compliance with data of questionable accuracy [5,6].

In the last 5 years, there have been at least 10 systematic reviews focused on EMA and/or reporting aspects of compliance to EMA schedules in youth (<18 years [7-9]; <22 years [10]), mixed youth and adult cohorts [11-13], or specific adult populations [5,14-16]. Compliance with EMA in youth (nonclinical and clinical samples) has been reported to range between 44% and 96% [8-10] and in mixed youth and adult cohorts, between 23% and 94% [11-14]. Reports of compliance in specific adult clinical populations range from 21% to 99% (chronic pain, 21%-99% [15]; psychotic disorders, 78%-86% [16]; substance use, 75%, (95% CI 72.37-77.65) [5].

Although Stone and Shiffman [17] have highlighted the need for explicit reporting of compliance in their original reporting guidelines for EMA, recurring issues relating to the reporting of compliance include (1) missing, incomplete, or ambiguous data; (2) heterogeneity in reporting; (3) impact of data exclusions; and (4) combining traditional (paper-based) and mEMA data [5]. Participant compliance with mEMA or mEMI—in theory—is related to the total protocol burden, which is a function of monitoring duration, frequency and complexity of prompts, and familiarity with the technology. However, as Jones et al [5] note, to date, there is little compelling, systematic evidence to support an association between EMA burden and compliance rates. These issues make it difficult to determine which, if any, features of EMA protocols positively or negatively influence compliance to EMA schedules.

The purpose of this systematic review is to guide the development of an mEMA protocol, which could be used for future studies of health-related behaviors and psychological constructs (including symptoms) in adults with and without chronic disease. The primary question for this systematic review is as follows: In adult nonclinical and clinical populations, which factors are associated with increased compliance to mEMA protocols for collection of health-related behaviors and psychological constructs (including symptoms)?

### Objectives

The objectives of this systematic review were to describe:

1. Health-related behaviors and psychological constructs assessed using mEMA
2. mEMA protocol and compliance reporting
3. Associations between key components of mEMA protocols and participant compliance

## Methods

**Search Registration**

The search strategy and review protocol were registered prospectively with the International Prospective Register of Systematic Reviews (PROSPERO 2016: CRD42016051726).

### Eligibility

Observational studies (cohort, cross-sectional) of mEMA in adults (>18 years of age) were eligible for inclusion in this review if these (1) reported participant compliance with mEMA; (2) were a primary study published in English between 2006 and 2016 inclusive; (3) included adults (≥18 years) either apparently healthy (nonclinical population) or with health conditions (clinical population); and (4) collected mEMA data using mobile devices as a primary or secondary outcome. References were excluded if these were (1) experimental designs investigating intervention efficacy; (2) duplicate publications or secondary analysis of the same data set; or (3) conference abstracts, protocols, commentaries (editorials or letters), or systematic or narrative reviews.

### Information Sources and Search Strategy

A range of electronic databases were searched to identify eligible studies: AMED (Allied and Complementary Medicine), CINAHL, Cochrane Library and CENTRAL (Cochrane Central Register of Controlled Trials), Embase, MEDLINE (including epub ahead of print), PsycINFO, Scopus, and Web of Science. An academic librarian (Carole Gibbs, University of South Australia) assisted with the development of the search strategy regarding conceptualization, operators (operational terms), and limiters [18] with the final search undertaken during a single week. Search terms and associated MeSH (Medical Subject Heading) alternatives, which were adapted for use in all databases, related to the population (adults), assessment (mEMA), and outcomes of interest (health behaviors, perceptual experiences including symptoms, affect or mood). Key search terms included “ecological momentary assessment,” “EMA,” “mobile ecological momentary assessment,” “mEMA,” “electronic diary,” “SMS or short message service,” “prompting,” “text messaging,” “health behaviour,” “symptom,” and “adult.” Reference lists of included studies and systematic reviews identified during the search were reviewed to identify additional potentially relevant studies.

### Study Selection

The titles and abstracts of studies identified from the search process were screened against a priori eligibility criteria and full-text versions imported into Covidence (Covidence systematic review software, Veritas Health Innovation). Both screening steps were undertaken by individual members of the research team working in pairs (AG and MW, HL and FF) with each person completing the task independently, before meeting with their partner to compare results and resolve disagreements (consensus).

### Data Collection

A data extraction template was prospectively developed; it was guided by the Checklist for Reporting EMA studies proposed by Liao et al [10] and pilot-tested on 5 randomly selected eligible studies. Working in pairs (AG and MW, JI and KF, HL and FF), individual members of the research team extracted all data before meeting with their partner to compare results and resolve disagreements by discussion. As this review aims to describe the features of mEMA schedules associated with increased mEMA protocol adherence, assessment of methodological bias was not planned.

#### Data Items

Data were extracted across 4 domains:

*Publication demographics:* title, authors, year of publication.

*Participants:* recruitment source, medical condition or diagnosis (clinical populations), sample size (enrolled, attrition or withdrawn and included in analysis), and age (mean/median, SD).

*mEMA protocol*: target behavior or psychological construct, mobile device type (PDA, palmtop computer, electronic diary, mobile or smartphone, tablet, other), participant training (yes/no), provision of incentives (course credit, financial, other, or none), incentive thresholds (yes/no) monitoring duration (days), prompt type (random signal, interval, event-based), frequency per day, number of questions/items per prompt type (reported or estimated from information reported in studies), strategy to deal with unanswered prompts, and time allowed for survey response. Where authors did not report the number of items per prompt type, but rather included descriptions of standardized instruments which were converted to mEMA survey items, a full version of the standardized instrument was accessed, and number of items calculated.

*mEMA compliance:* verbatim (or where possible calculated from reported data), participant completion (number included in analysis, data exclusions), criteria/thresholds for mEMA data, number of prompts delivered/answered per person/cohort (planned, actual, average, range), and response latency as time (mean, SD) [8,10].

#### Data Management

Data were tabulated to provide descriptive summaries. The mEMA surveys commonly included multiple questions reflecting behavioral or psychological constructs. Although the authors of mEMA studies did not always specify the primary outcome for these observational studies, most studies explicitly reported the key variable of interest for mEMA, which we interpreted to be the primary mEMA target. Where other data were also collected by the same mEMA survey, we denoted those as secondary mEMA targets. The primary mEMA target of studies was identified, and studies were grouped and reported according to two broad domains: (1) behavior (eg, dietary, physical activity, and smoking) and (2) psychological construct (eg, affect, cognition, and sensations/symptoms). For each domain, a narrative synthesis was used to summarize participants, mEMA protocol, and compliance data for nonclinical and clinical data sets.

With the exception of device type, where possible, we adopted the operationalization of variables common to Wen et al [9] or Jones et al [5] unless the distribution of our data resulted in very unbalanced cells or our data could provide greater resolution. Potential mEMA protocol factors related to compliance were categorized for analysis. *Monitoring duration* was categorized as follows: <7 days, >7 days to <14 days, or >14 days. *Prompt frequency* was grouped as follows: 1-3 prompts per day; 4-5 prompts per day; or ≥6 prompts per day. Minimum *items per prompt* were categorized as follows: ≤5, >5 to ≤9.5, >9.5 to ≤26, and >26. *Device type* was categorized as mobile phone, PalmPilot/PDA, or other. The reporting of *training or familiarization sessions or provision of incentives* were dichotomized as yes/no or labeled as not reported.

Given ongoing concerns about the burden imposed by EMA schedules and compliance, in addition to these individual factors, we explored a novel composite metric to reflect aspects previously identified as possible contributing factors (monitoring duration, frequency, type, and complexity of prompts).

Where possible, a mEMA *burden score* was calculated for each study by multiplying:

- the total monitoring duration in days (d; all days included in all waves)
- by the maximum frequency of time-based prompts (random and interval) per day (f)
- by the minimum number of compulsory questions/items within all prompts per day (i) and
- by a weighting reflecting the number of prompt types scheduled per day (w; eg, time-based [signal or interval] and/or event-based) with each prompt type weighted as 1 (min weight=1, max=3).

For example, the mEMA burden score for a 14-day monitoring schedule *(d),* where 5 random signal prompts were delivered per day (*f*), with each prompt requiring responses to a minimum of 12 items/questions (*I*; 60 items in total per day), would be 840. If event-based prompts (irrespective of the number of items within the prompt) were added to this schedule (*w*), the burden score would rise to 1680. *Burden scores* were calculated and reported in quartiles: 0 to 283.5, 284 to 810, 811 to 1806, or ≥1807.

### Meta-analysis

Random effects restricted maximum likelihood estimator meta-analyses were undertaken using the approach reported by Jones et al [5] and Wen et al [9], with both authors advising to assist in accurate replication. All statistical analyses were conducted using JASP (Jeffreys's Amazing Statistics Program, version 0.9.2; 2019). Studies were included in the meta-analysis if they reported all data necessary for the meta-analysis procedure and cohort compliance (%) could be extracted before data exclusions when possible. Sensitivity analysis was conducted to explore the impact of compliance rates reported before and after data exclusion. The effect sizes (ESs) were calculated by logit transforming the proportion of completed prompts (ie, compliance rates; proportion/[1−proportion]). SEs were then estimated using the following equation:

√([1/np]+[1/n{1−p}])

Where, *n* is the sample size and *p* is the proportion.

To adjust for clustering within participants, the SE was adjusted by the effective sample size (ESS). The ESS equation is as follows:

kn/(1+[k−1] ICC)

Where, k is the number of study prompts, n is the participant number, ICC is either the reported intraclass correlation coefficient (ICC) or the SD of reported compliance, and p is the proportion of completed prompts.

For studies that did not report SD data, sensitivity analyses were conducted by computing the SEs using the 25 and 75 percentiles of available SDs. The sensitivity analyses did not show any differences. Therefore, analysis used imputed median SD (where the original SD was not reported). To aid interpretation, inverse logit transformation was conducted to enable reporting of proportions. The I^2^ statistic was used to quantify heterogeneity across the ES. Pooled compliance rates were initially explored for combined nonclinical and clinical data sets and then compared between nonclinical and clinical studies.

To explore the relationships between the pooled compliance rates (nonclinical and clinical data sets) and EMA protocol factors (ie, monitoring duration, prompt frequency, device type, training, incentives, and burden score), random effects analysis of variance was conducted as part of the meta-analysis program. Moderator analyses were conducted separately for nonclinical and clinical pooled compliance.

## Results

### Overview

**Figure 1:**
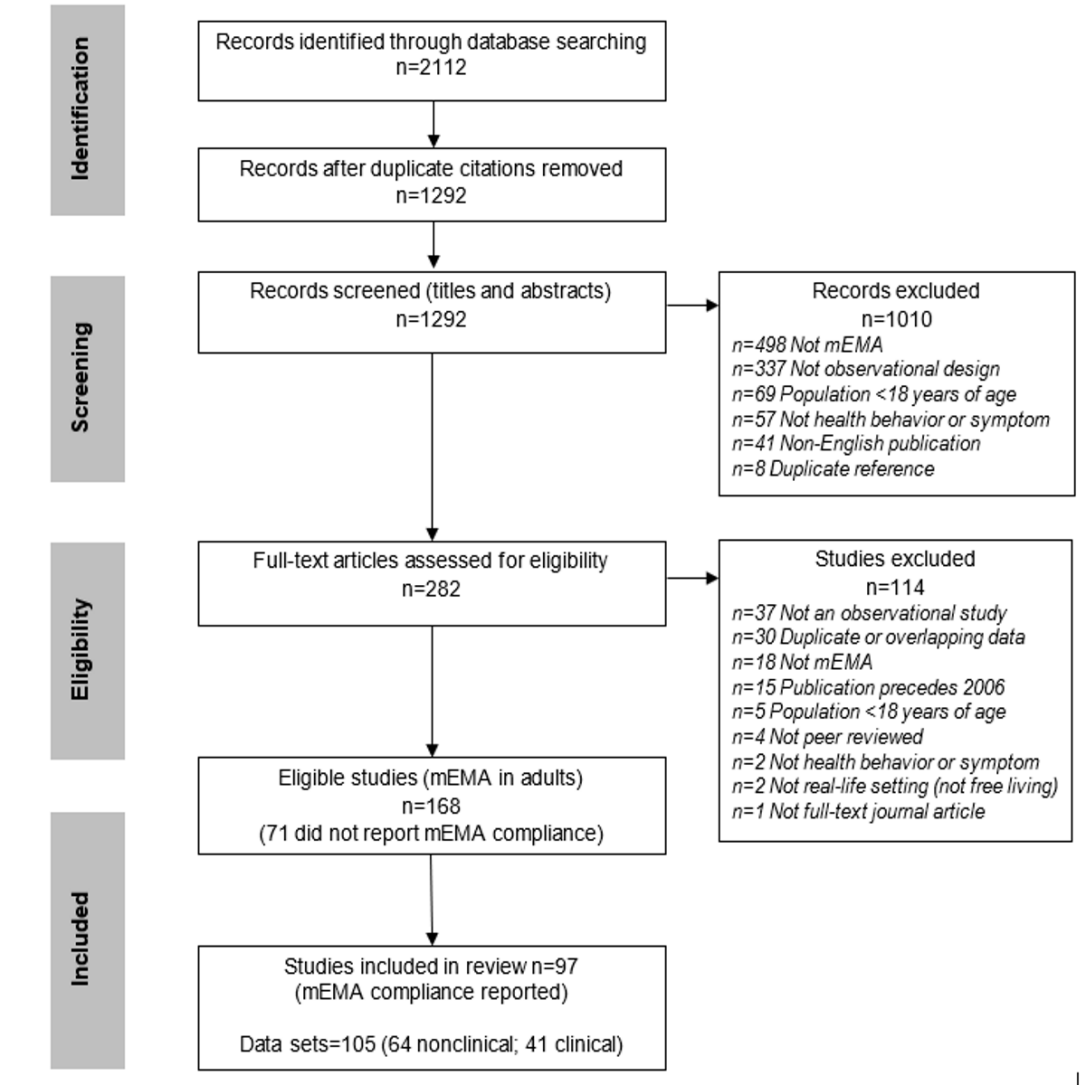
Search strategy process and final outcomes (hand searching of reference list-eligible studies and review papers did not identify additional studies to those returned by database searches). mEMA: mobile ecological momentary assessment.

Figure 1 presents the outcome of the search strategy. Of the 282 studies reviewed as full text, 168/282 (59.6%) included mEMA; however, 42.3% (71/168) were excluded because mEMA compliance was not reported. The majority of the 97 studies retained for this review comprised studies that recruited or reported a single nonclinical group (61/97, 63%) or a clinical (31/97, 32%) group. Two studies included 2 [19] or 3 clinical groups [20]. In addition, 3 studies included clinical and nonclinical comparator groups (4 groups [21], 2 groups [22,23]). Overall, 105 data sets were included in this review (nonclinical: 64/105, 61%; clinical: 41/105, 39%). A description of all included data sets is presented in Multimedia Appendix 1 [19-114].

A total of 44,796 participants were included in the analyses (nonclinical: 42,338/44,796, 94.51%; clinical: 2431/44,796, 5.43%) with a median sample size of 62 (nonclinical: n=89; clinical: n=40; Multimedia Appendix 2). Two data sets (nonclinical) were outliers because of the sample size (n=21,947; n=11,572) [24,25]. The main sources of recruitment for nonclinical data sets were educational institutions (30/64, 47%) and community (26/64, 41%), whereas clinical data sets were predominantly recruited from medical/health services (21/41, 51%) and community (17/41, 41%). For clinical data sets, the most common health conditions were psychiatric or mental health (12/41, 29%), chronic pain and fibromyalgia (6/41, 15%), and eating disorders (5/41, 12%). Multimedia Appendix 2 presents a summary of the study characteristics grouped by primary mEMA target.

### Objective 1: Health-Related Behaviors and Psychological Constructs Assessed With mEMA

Using the primary mEMA target, data sets were grouped into 2 broad domains: *Behavior* or *Psychological construct*. Within the *Behavior* domain, the *Other* category reflects single studies (7), where the primary mEMA target did not align with more common behavior targets (social interactions/activities [26,27], sexual [28], leisure [29], nonsuicidal self-injurious [30], HIV prevention [31], and oral behaviors) [32].

The most frequent primary mEMA target across all domains for nonclinical and clinical data sets was affect (31/105, 29.5% of data sets; nonclinical n=15/64, 14%, clinical n=16/41,15%). The most common primary mEMA target in nonclinical data sets (n=64) reflected the Behavior domain (total 38/64, 59%), whereas clinical data sets (n=41) reflected the Psychological domain (total 32/41, 78%).

With the exception of 1 clinical study (fatigue) [33], the remaining data sets included mEMA items/questions beyond the primary mEMA target. The most frequent secondary targets assessed were affect (50/105, 47.6%), social environment (33/105, 31.4%), physical activity (25/105, 23.8%), cognition (24/105, 22.8%), and physical environment (20/105, 19%). Multimedia Appendix 2 presents a summary of secondary mEMA targets and participant characteristics grouped by the primary mEMA target.

### Objective 2: mEMA Protocol and Compliance Reporting

Multimedia Appendix 3 presents a summary of mEMA protocols grouped by primary mEMA target. Among the included studies, mEMA data were most commonly collected using handheld computer/PDAs (61/105, 58.1%) with mobile phones accounting for approximately one-third (37/105, 35.2%). Participant training in mEMA was reported by most studies (nonclinical: 49/64, 77%; clinical: 35/41, 85%). The provision of incentive (financial or other) was more frequent in nonclinical protocols (nonclinical: 46/64, 72%; clinical: 20/41, 49%).

Across all data sets (n=105), the median monitoring duration for mEMA protocols was 7 days (range: 1-182 days), with durations differing between nonclinical (median 7 days, range 1-49 days) and clinical protocols (median 12 days, range 1-182 days). Most studies included a single prompt type (overall data sets: 69/105, 65.7%; nonclinical: 40/64, 63%; clinical: 29/41, 71%), with random signals being the most common in nonclinical protocols (49/64, 77%) and interval in clinical protocols (25/41, 61%). Of the remaining study protocols, 23% (24/105) of studies included 2 prompt types and 11% (12/105) protocols included all 3 prompt types (random signal, interval, and event-based). The frequency of time-based prompts (signal or interval) ranged from 1 to 42 per day (median: nonclinical=5, range 1-36; clinical=4, range=1-42). The number of specific questions/items within a standard prompt varied markedly across study protocols; it ranged between 1 and 73 (median: nonclinical=10; clinical=8).

Table 1 presents a summary of reporting for compliance metrics for mEMA time-based prompts (ie, signal and fixed prompts). Participant attrition (dropout*)* rates were reported or could be calculated for half of the 105 data sets (nonclinical: 31/64, 48%; clinical: 22/41, 54%). Less than half of the data sets reported the number of prompts delivered (overall: 22/105, 21%; nonclinical: 14/64, 22%; clinical: 8/41, 20%) or answered (overall: 43/105, 41%; nonclinical: 29/64, 45%; clinical: 14/41, 34%). Approximately one-third of the data sets reported a criterion for valid mEMA data or reasons for data exclusions (overall: 37/105, 35%; nonclinical: 25/64, 39%; clinical: 12/41, 29%). Criteria for valid EMA data fell into 2 main groups, with the most common based on assessment completion (ie, specified threshold for number of prompts completed per day or percentage of overall compliance), followed by response latency period threshold (eg, prompt required to be answered within 30 min). Of the data sets reporting a criterion for response time (overall: 38/105, 36%; nonclinical: 16/64, 25%; clinical: 22/41, 54%), this ranged from 1.5 to 60 min (median 15 min; Multimedia Appendix 3). Other reasons for data exclusion were based on specific time of day prompts (excluding the first or last of the day), technical malfunctions, or unspecified (eg, general statements on participants’ limited or poor compliance).

Of the 105 data sets, 82/105 (78.1%) reported compliance using a single metric (cohort, average per person or other), with compliance at the cohort level most common (overall: 62/105, 59%; nonclinical: 34/64, 53%; clinical: 28/41, 68%). Compliance was less frequently reported using the single metric of average per person (overall: 20/105, 19%; nonclinical: 14/64, 22%; clinical: 6/41, 15%) or compliance for both cohort and average per person (overall: 18/105, 17%; nonclinical: 12/64, 19%; clinical: 6/41, 15%). The remaining data sets (n=5; nonclinical: n=4, clinical: n=1) reported compliance after combining event/time-based signals [34] or separate tasks [35], number of completed protocol days [36], total number of prompts (data) available [37], or proportion of completed questions/items per prompt [38]. Cohort compliance reported before data exclusions ranged from 38% to 98% (median 82%) and after data exclusions from 50% to 97% (median 81%; Table 1).

**Table 1:** Summary of mobile ecological momentary assessment (mEMA) compliance reporting.

Primary mEMA^a^ target	NC^b^ or C^c^ (n)	Reported N=data sets (%)							Cohort compliance (%)	
		Attrition rate	Total prompts delivered	Total prompts answered	Criteria for valid data	Compliance predata exclusions	Compliance postdata exclusions	Average per-person compliance	Predata exclusion, median (range)	Postdata exclusion, median (range)
Smoking	NC (12)	8 (66)	4 (33)	5 (42)	4 (33)	7 (58)	1 (8)	5 (42)	83 (69-93)	83 (74-91)
	C (1)	1 (100)	1 (100)	1 (100)	0 (0)	1 (100)	0 (0)	1 (100)	68 (NA^d^)	N/A^e^
Alcohol	NC (8)	3 (37)	1 (12)	3 (37)	3 (37)	4 (50)	4 (50)	2 (25)	90 (86-97)	79 (69-80)
	C (0)	N/A	N/A	N/A	N/A	N/A	N/A	N/A	N/A	N/A
Eating behaviors	NC (10)	6 (60)	2 (20)	5 (50)	5 (50)	4 (40)	3 (3)	5 (50)	90 (40-96)	67 (50-71)
	C (3)	2 (66)	1 (33)	2 (66)	1 (33)	2 (66)	1 (33)	0 (0)	N/A	78 (68-87)
Physical activity	NC (5)	1 (20)	1 (20)	4 (80)	0 (0)	3 (60)	0 (0)	3 (60)	82 (75-95)	N/A
	C (1)	0 (0)	0 (0)	0 (0)	0(0)	1 (100)	0 (0)	0 (0)	N/A	97 (NA)
Other	NC (3)	0 (0)	2 (66)	3 (100)	0 (0)	2 (66)	0 (0)	2 (66)	61 (38-84)	N/A
	C (4)	4 (100)	0 (0)	3 (75)	1 (25)	2 (50)	1 (25)	2 (50)	74 (72-74)	N/A
Personality traits	NC (7)	4 (57)	1 (14)	3 (42)	3 (42)	3 (42)	1 (14)	3 (42)	75 (55-90)	N/A
	C (0)	N/A	N/A	N/A	N/A	N/A	N/A	N/A	N/A	N/A
Affect	NC (15)	7 (46)	2 (13)	5 (33)	9 (60)	6 (40)	6 (40)	7 (46)	78 (63-90)	77 (73-81)
	C (16)	9 (56)	2 (12)	5 (31)	7 (44)	5 (31)	6 (37)	9 (56)	80 (69-96)	83 (79-87)
Cognitions	NC (2)	1 (50)	0 (0)	1 (50)	0 (0)	2 (100)	0 (0)	0 (0)	83 (77-89)	N/A
	C (0)	N/A	N/A	N/A	N/A	N/A	N/A	N/A	N/A	N/A
Symptoms	NC (2)	1 (50)	1 (50)	0 (0)	1 (50)	0 (0)	1 (50)	1 (50)	N/A	N/A
	C (16)	6 (37)	4 (25)	3 (19)	3 (19)	11 (69)	4 (25)	4 (25)	90 (68-98)	86 (86-93)
Total	NC (64)	31 (48)	14 (22)	29 (45)	25 (39)	31 (48)	16 (25)	28 (44)	82 (38-97)	74 (50-91)
	C (41)	22 (54)	8 (20)	14 (34)	12 (29)	22 (54)	12 (29)	16 (39)	80 (68-98)	87 (68-97)
	*t* (105)	53	22	43	37	53	28	44	82 (38-98)	81 (50-97)
	%	50.4	20.9	40.9	35.2	50.4	26.6	41.9	N/A	N/A

^a^mEMA: mobile ecological momentary assessment.

^b^NC: nonclinical.

^c^C: clinical.

^d^NA: not available as domain includes a single study.

^e^N/A: not applicable.

### Question 3: Associations Between Key Features of mEMA Protocols and mEMA Compliance

Of the 105 data sets included in this review, 65% reported sufficient data for inclusion in the meta-analysis (n=68 data sets: 41/105 [39%] ES nonclinical and 27/105 [26%] ES clinical); Multimedia Appendix 1) [20,21,23,26,27, 29-31,33,36,39-90]. The remaining data sets did not report cohort compliance but reported average per-person compliance [19,24,25,91-106,28,32] or other [34,35,37,38,107-110], or where cohort compliance was reported, a variable required for the meta-analysis was not [111-114].

The overall compliance rate across all 68 ESs was 81.9% (95% CI 79.1-84.4). There was sizable heterogeneity across the compliance rates (I^2^=98). Sensitivity analysis exploring the impact of pre and postdata exclusion compliance rates showed no significant difference (*P*=.67*;* before exclusion: n=50, 81.6%; after exclusion: n=18, 82.8%). There was no significant difference (*P*=.16) between the pooled compliance of nonclinical studies (80.4%; 95% CI 76.1-83.9; I^2^=98.6) and clinical studies (84.2%; 95% CI 80.1-87.4; I^2^=95.7). Three studies included more than 1 data set and reported compliance ESs for each (data sets n=2 [23], n=3 [20], and n=4 [21]). Sensitivity analysis was undertaken to explore the impact of double counting of mEMA protocol factors within the meta-analysis, where multiple ESs were reported within single studies. When a single ES was retained for each of these studies (lowest ES of the 2 [23], median of 3 [20], ES closest to the average for 4 [21]), the pooled 62 ESs (81.3%, 95% CI 78.2-84.2) and reported variance (I^2^=98) were essentially the same as the full data set (68 ESs: 81.9%; 95% CI 79.1-84.4; I^2^=98). To ensure that subgroup analysis was not affected, all analyses were conducted without duplicate ESs, and all relationships were consistent with those of the full data set.

For nonclinical studies, 2 factors (prompt frequency and items/prompt) were significantly related to mEMA compliance. For prompt frequency, the overall model was nonsignificant (*P*=.07), but the coefficient was significant (*P*<.001). Prompting 1 to 3 times per day was associated with higher compliance (87%; 95% CI 82.5-90.4) compared with studies with more than 3 prompts per day (76.9%) and 6 or more prompts per day (79.4%). The number of items per prompt was significant for both the overall model (*P*=.04) and the coefficient (*P*<.001). Factor analysis showed that prompts with more than 26 items had significantly lower compliance (63%; 95% CI 42.3-79.7) compared with prompts with ≤26 items (categories: ≤5; >5 to ≤9; >9.5 to ≤26; compliance range: 84%-78.6%).

For clinical data sets (n=27), no factors were significantly related to compliance. The number of items per prompt approached significance (*P*=.05). Compliance appeared to be lower in studies with 9.5-26 items per prompt (71.1%; 95% CI 62.5-78.6). Significant heterogeneity was reported for all significant findings (nonclinical and clinical), with I^2^ values in excess of 90%, suggesting that although some variance can be explained by the significant factors, a large amount of variance remained unexplained. The burden score was not significantly related to compliance. The meta-analysis factor analysis compliance proportions are presented in Table 2.

**Table 2:** Meta-analysis results for clinical and nonclinical data sets.

Characteristics		Clinical data sets, n=27		Nonclinical data sets, n=41	
Protocol factors		n (%)	Pooled compliance (95% CI)	n (%)	Pooled compliance (95% CI)
**Monitoring period, day**					
	<7	12 (44)	81.6 (74.1-87.3)	24 (58)	77.4 (71.3-85.5)
	>7 to ≤14	4 (15)	84.4 (74.3-91.1)	9 (22)	82.1 (71.30-89.5)
	>14	11 (41)	86.7 (81.2-91.0)	8 (19)	85.3 (80.5-89.1)
**Device^a^**					
	Mobile	5 (19)	88.6 (71.5-96.1)	17 (41)	78.6 (71.9-84.0)
	PDA	18 (66)	81.9 (77.4-85.8)	22 (54)	80.2 (74.2-84.9)
	Other	4 (15)	88.8 (82.4-93.1)	2 (5)	92.2 (86.3-95.7)
**Training**					
	Yes	23 (85)	84.4 (79.7-88.4)	36 (88)	80.4 (76.0-84.3)
	No	0 (0)	N/A^b^	0 (0)	N/A
	NR^c^	4 (15)	82.8 (78.4-86.4)	6 (15)	77.7 (73.1-82.0)
**Incentives**					
	Yes	13 (48)	83.6 (77.7-88.3)	35 (85)	80.4 (79.0-84.3)
	No	0 (0)	N/A	6 (15)	77.9 (73.1-82.0)
	NR	18 (66)	85.7 (81.3-89.3)	0 (0)	N/A
**Prompt frequency, per day**					
	1-3	8 (30)	85.3 (77.6-90.7)	8 (19)	87.0 (82.5-90.4)
	4-5	12 (44)	81.5 (75.8-85.9)	16 (39)	76.9 (70.1-82.5)
	≥6	6 (22)	86.3 (74.1-92.4)	17 (41)	79.4 (71.1-85.5)
	UTD^d^	1 (4)	90.6 (N/A)	0 (0)	N/A
**Burden score**					
	0-283.5	4 (15)	86.2 (76.9-92.4)	11 (27)	80.5 (75.7-84.6)
	284-810	7 (26)	86.4 (75.4-93.0)	10 (24)	79.6 (73.7-84.7)
	811-1806	3 (11)	88.8 (64.8-97.1)	13 (31)	82.8 (73.7-89.1)
	≥1807	7 (26)	85.3 (80.5-89.0)	4 (10)	79.1 (51.5-93.1)
**Items per prompt**					
	<5	8 (30)	87.2 (80.7-91.9)	10 (24)	82.8 (77.2-87.2)
	5 to ≤9.5	7 (26)	88.4 (76.9-94.6)	8 (19)	78.6 (67.5-86.8)
	9.5 to ≤26	2 (7)	71.1 (62.5-78.6)	16 (39)	84.0 (79.0-88.0)
	>26	6 (22)	87.2 (82.9-90.7)	4 (10)	63.0 (42.3-79.7)
	NR	5 (19)	72.7 (68.4-76.9)	3 (7)	70.3 (40.4-89.2)
**Number of prompt types**					
	1	18 (66)	82.6 (78.1-86.5)	25 (61)	79.6 (75.0-83.5)
	2	6 (22)	86.4 (71.3-94.2)	11 (27)	83.3 (71.7-90.9)
	3	3 (11)	87.2 (85.5-88.8)	5 (12)	77.7 (65.7-86.5)

^a^Device type included with categories: Mobile phone (total n=22; smartphone: clinical n=1; nonclinical n=14; mobile: clinical n=4, nonclinical n=3); PDA (total n=45; clinical n=22, nonclinical n=23); Other (total n=6; electronic diary: clinical n=2, nonclinical n=1; iPod: clinical n=1, nonclinical n=1; watch device: clinical n=1).

^b^N/A: not applicable.

^c^NR: not reported.

^d^UTD: unable to be determined.

## Discussion

### Principal Findings

This systematic review of observational studies aimed to describe protocols and compliance with mEMA for self-reported health-related behaviors and psychological constructs in adults. Across 105 unique data sets, the key findings of this review were as follows: (1) a variety of health-related behaviors and psychological constructs were assessed, with affect being the most common mEMA target; (2) mEMA protocols varied widely across studies; (3) compliance was inconsistently reported across studies; (4) meta-analysis estimated an overall compliance rate of 81.9% (95% CI 79.1-84.4), with no significant difference between nonclinical and clinical data sets or estimates before or after data exclusions; (5) compliance was associated with prompts per day and items per prompt (nonclinical); and 6) no compelling relationship was identified between key features of mEMA protocols representing *burden* and mEMA compliance.

### mEMA Use in Adults for Health-Related Behaviors and Psychological Constructs

The mEMA targets identified in this review reflect those reported in previous systematic reviews: affect/mood [7,12,14,15], cognitions [13], symptoms [15], eating or dietary behaviors [10,11], physical activity [10], and smoking or alcohol consumption [5,6]. Likewise, clinical populations identified in this review (psychiatric or mental health conditions, chronic pain and fibromyalgia, eating disorders, and substance use) were generally consistent with those reported previously [5,7,11,12,14-16]. However, there were chronic conditions unique to this review: oral or dental health, cancer, stroke and traumatic brain injury (for each n=3, 9/41, 22%), HIV, and upper abdominal surgery (for each n=1, 2/41, 5%). The small number of studies identified for these clinical groups may suggest that the potential for mEMA has not yet been realized in these populations.

### Reporting of mEMA Protocols and Compliance

Most studies included in this review provided information around the EMA protocol used (device, monitoring duration, frequency and type of prompts, provision of training, and use of incentives). Consistent with previous systematic reviews of both youth and adults, there was considerable heterogeneity across studies for EMA protocols (Multimedia Appendix 3). Heterogeneity may be expected given the various potential applications of this survey approach. The mEMA protocol required to obtain sufficient or appropriate self-reported data on daily habitual behaviors in the general population is not likely to be the same as that for obtaining self-reported data on psychological responses to events or stimuli in clinical contexts. For example, the average EMA monitoring duration for studies of nonclinical adults in this review was 7 days (range: 1-49 days) compared with 12 days (range: 1-182 days) for clinical populations and 30 days (range: 3-730 days) in a review of EMA in substance users [5]. Likewise, prompt type, frequency, and complexity are expected to differ depending on the EMA target and population. Reviews of studies of EMA for diet and physical activity (common behaviors) report a daily average prompt frequency of 20 [10] compared with less than 4 prompts per day in substance use [5]. For these reasons, in systematic reviews of EMA use—including this one—reporting of summary metrics (mean, SD, median, range) for protocol components could be interpreted as a reflection of diversity in EMA application rather than a lack of protocol standardization.

The same rationale cannot be applied to the inconsistencies identified in reporting of EMA protocol compliance. Compliance is problematic to determine for event-based prompts (eg, those completed with smoking or consumption of alcohol). Compliance for time-based notifications, especially when the EMA is conducted using mobile devices, is relatively simple (number of prompts answered out of the total number of prompts delivered). However, participants may respond to a notification but may not complete all survey items or may not respond in a timely manner, affecting the momentary aspect of the EMA. In both of these cases, the act of responding might appropriately contribute to compliance rates, but the data are unlikely to be valid. These concepts were evident in the earliest recommendations for reporting compliance in EMA studies [17], which predate the sampling frame of this systematic review (2006-2016 inclusive). Considering that 71 studies were excluded from this review because of the absence of reporting mEMA compliance, less than half of the studies included in this review complied with recommendations put forward by Stone and Shiffman [17], such as reporting the proportion of delivered prompts answered (43/105, 41%) or defining a criterion for valid EMA data (37/105, 35%). Similarly, less than half of the data sets included in this review reported an average number of prompts answered per person (44/105, 42%), as recommended by more recently published guidelines for reporting EMA [8,10].

With the growth of systematic review methodologies (meta-synthesis, meta-regression, etc), one aspect of reporting for EMA warrants further consideration. EMA allows collection of self-report data across multiple survey items reflecting a range of behavioral, psychological, and contextual factors. It is not uncommon for data collected in the original, primary study to be reported in several publications. The foci of these *offspring* publications may include the total original sample of participants recruited (eg, unpublished data for specific mEMA items or other variables) or explore a subset of the original study participants (eg, patterns associated with participant characteristics). Although this is a reasonable and defensible use of the original study’s resources, identification of duplicate or overlapping data in studies can be problematic. Where ambiguity exists, contacting the study authors is one way to clarify which publication should be considered the primary report (and which report overlapping or duplicate data). However, this option becomes less practical as time and people move on. The alternative is for authors to include an explicit statement concerning the existence of publications that include overlapping or duplicate data. There were a number of exemplars of this aspect of reporting in studies included [67,68,96] and excluded from this review [115-118].

### Associations Between Key Components of mEMA Protocols and Compliance: Meta-analysis

In our meta-analysis (68 data sets), which replicates and was guided by the authors of 2 previous meta-analyses on this topic [5,9], the overall compliance rate was 81.9% (95% CI 79.1-84.4). This was slightly higher than that reported by Wen et al [9] (78.26%; 95% CI 75.49-80.78) and Jones et al [5] (75.06%; 95% CI 72.37-77.65). Although concerns have been expressed about the relationship between EMA burden and compliance, it remains unclear whether, or which, EMA protocol factors affect participant compliance. In our meta-analysis, for nonclinical data sets, prompt frequency per day and the number of items per prompt were significantly related to compliance (noting that it is not unusual for coefficients derived within a model to be significant even when the overall model is not). However, the findings are likely affected by the number of data sets in some categories. For nonclinical data sets, frequencies of 1-3 prompts per day were associated with small but significantly higher mean cohort compliance. Higher compliance with lower number of prompts perhaps seems intuitive, yet the evidence is inconsistent. Wen et al [9] reported opposite patterns of significance when nonclinical and clinical population data were investigated, and Jones et al [5] and Ono et al [119] reported no relationship with prompt frequency and compliance among substance users and those affected by chronic pain, respectively.

The relationship between the number of items included within each prompt and compliance has not been explored in previous systematic reviews or meta-analyses of mEMA. In this review, the number of items respondents were required to complete in a standard prompt ranged from 1 to 73 (median 10), with a greater number of items more common in the mEMA of psychological constructs (Multimedia Appendix 3). Our analysis showed an intuitive relationship with compliance among nonclinical data (ie, ≥26 items per prompt had the lowest mean cohort compliance of 63%; 95% CI 42.3-79.7), but not with clinical data.

When aiming to identify protocol factors affecting compliance, the inconsistencies in reporting of EMA compliance and the likely publication bias (studies with lower compliance rates may not be submitted or accepted for publication) must also be considered [5]. These factors limit the inclusion of potentially eligible studies in meta-analyses (68/105, 64.8% data sets in this review; 36/42, 86% studies in a previous review [9]). In addition, studies included in meta-analyses privilege *best compliers* through exclusion of participants not meeting criteria for valid EMA data or compliance thresholds (determined a priori or posteriori). Jones et al [5] attempted to address this latter point by exploring protocol factors associated with participant data exclusions (monitoring duration and prompt frequency). Finally, aggregate level compliance may not be sensitive enough or provide sufficient resolution to identify factors associated with higher or lower compliance. While accepting these caveats, there are 2 ways to consider the results of the 3 meta-analyses undertaken by Wen et al [9], Jones et al [5], and this study:

1. There is insufficient resolution to identify associations—if they exist—at the aggregate data level.
2. Although confidence limits might be reduced by adding further studies, the meta-analyses are essentially correct, and the notion of protocol burden imposed on participants has little to no impact on compliance [4,5].

In studies using EMA, the issue of what constitutes an acceptable rate of compliance or missing data is debatable. Although several studies included in this review cite a criterion or commonly used threshold of 80%, we, similar to Jones et al [5], could not identify the derivation of this criterion. For authors currently planning, conducting, or writing papers or protocols on EMA to monitor health-related behaviors of psychological constructs, adequate recording and reporting of compliance data following recommendations by Liao et al [10] and Heron et al [8] should enable future meta-analyses to explore protocol factors affecting participant compliance rates.

This systematic review prospectively aimed to sample a decade of mEMA use (protocol registered in November 2016; sampling frame of 2006 to 2016) in observational studies including adults from clinical and nonclinical populations. As one of the first EMA reporting documents was published in 2002 [17], this sampling frame assumed that researchers planning or reporting studies including mEMA would be aware of these reporting recommendations. The time frame required for the uptake of EMA reporting recommendations is unknown, although estimates of the time required for uptake of translational research ranges between 2 and 17 years [120]. Our sampling frame and review, however, does not capture studies published from 2017 to date. It is possible that more recent publications differ from those included in our review (greater mobile phone use, better reporting of mEMA schedules, and compliance).

There are no universally accepted recommendations concerning the updating of systematic searches or incorporation of the newer studies into the review results. Systematic reviews—depending on the specific question and volume of studies eligible for inclusion—are time- and labor-intensive. For larger reviews, it is not uncommon for these to take >2 years [121], with updates of Cochrane Collaboration systematic reviews taking up to 3.3 years [122]. The current Cochrane Collaboration policy infers that the decision to update a systematic review should consider the importance of the review question and the volume of new information (studies) [122]. Early in the review process (postsearch completion), 2 papers were identified, published in 2016 [10] and 2017 [8], providing updated recommendations for EMA reporting. Although the volume of mEMA studies published from 2017 is substantial and growing, we opted not to undertake an updated search/meta-analysis to *quarantine* mEMA studies published before the availability of the more recent EMA reporting recommendations.

### Strengths and Limitations

This review was strengthened by the broad eligibility criteria used, including studies across nonclinical and clinical contexts in adults. The meta-analysis method was replicated from previous studies [5,9], enabling direct comparison of findings. To the best of the authors’ knowledge, this review is the first to propose and explore *burden* as a compound effect of the various EMA factors (monitoring duration, prompt frequency and prompt type, item per prompt) on participant compliance. We have proposed this novel metric as a starting point for conversations, critique, and further development. In its current form, the burden metric does not include all factors likely to contribute to burden (unfamiliarity with technology, adjunctive use of wearable technologies such as accelerometers), the proposed weighting is rudimentary, and the accuracy of study design features was not confirmed by the study authors.

Limitations of this review include a search strategy focused on the use of mEMA and excluding interventions delivered using EMA (EMI). Consequently, the findings of this review should not be extrapolated or assumed to be similar in studies using EMI. Most studies included in this review provided a clear statement of the primary outcome of interest within each observational study, and we are confident that our categorization of primary mEMA targets is defensible. However, when observational studies did not clearly identify or infer a primary outcome of interest and given mEMA survey items can include multiple items for both self-reported behavioral and psychological constructs, for a small number of studies, misclassification may exist with respect to categorization of mEMA targets as primary or secondary. In the absence of explicit statements by the authors on the number of items within each standard notification, we adopted a conservative approach by estimating the minimum compulsory number of items based on either the information provided by authors within publications or reviewing the instruments reported by authors for inclusion within surveys. The impact of including only studies published in English is unknown.

### Conclusions

This review suggests that there is substantial interest in the use of mEMA in adults to collect self-reported health-related behavior and psychological construct data in nonclinical and clinical contexts. Across mEMA studies, there was considerable heterogeneity in protocol design, which may reflect a concerted effort by researchers to tailor mEMA protocols for the intended target and/or population to facilitate compliance. However, the number of studies reporting participant compliance with EMA is concerning. As a result of no or underreporting of compliance, pooled compliance rates may be skewed in favor of overall higher EMA compliance rates. This may dampen associations between compliance rates and EMA protocol factors or burden, making it difficult to ascertain which, if any, protocol factors (such as prompt frequency and number of items within prompts, as identified in this analysis) improve compliance and data collection.

## Appendix

Multimedia Appendix 1 Ecological momentary assessment (EMA) population and compliance characteristics for studies included within review.

Multimedia Appendix 2 Summary of mobile ecological momentary assessment (mEMA) targets and participant characteristics in nonclinical and clinical mEMA studies.

Multimedia Appendix 3 Summary of mobile ecological momentary assessment protocols.

## Abbreviations

EMA: ecological momentary assessment
ES: effect size
ESS: effective sample size
mEMA: mobile ecological momentary assessment
mEMI: mobile ecological momentary intervention
