# Supplementary material for: Compliance With Mobile Ecological Momentary Assessment of Self-Reported Health-Related Behaviors and Psychological Constructs in Adults: Systematic Review and Meta-analysis
Source: J Med Internet Res. 2021 Mar 3;23(3):e17023. doi: 10.2196/17023 (PMC7970161; doi:10.2196/17023)
Supplement: Multimedia Appendix 1 [file jmir_v23i3e17023_app1.docx]

| **Multimedia Appendix 1**  **Table 1** Ecological momentary assessment (EMA) population characteristics for studies included within review (ordered by first author surname in nonclinical and clinical populations). | | | | | | | | |  |
| --- | --- | --- | --- | --- | --- | --- | --- | --- | --- |
| **Study** | | | **Population** | | | | | |  |
| ^a^ = Included in MA | |  | Clinical (C)/ Nonclinical (NC) | Clinical population | Primary mEMA target | Sample size n= | Sampling source | Age (mean [SD] range)  (years) | |
| ^a^ | Ambwani et al. [39] | 2015 | NC | - | EAT. | 40 | EI | 19.22 [1.35] 18-23 | |
| ^a^ | Andersson et al. [40] | 2007 | NC | - | ALC. | 15 | EI | 26.5 [4.0] NR | |
| ^a^ | Armey et al. [30] | 2011 | NC | - | MIS. | 36 | EI | 18.7 [0.7] NR | |
|  | Asselbergs et al. [91] | 2016 | NC | - | AFF. | 27 | EI | 21.1 [2.2] 18-25 | |
| ^a^ | Berg et al. [41] | 2014 | NC | - | EAT. | 50 | Com | 43.0 [11.0] 21-64 | |
| ^a^ | Buckner et al. [42] | 2012 | NC | - | AFF. | 49 | EI | 19.14 [1.0] 18-22 | |
|  | Burns et al. [108] | 2015 | NC | - | SYM. | 105 | M | 45.96 [13.2] NR | |
| ^a^ | Burt et al. [43] | 2010 | NC | - | PER. | 103 | EI | 19.5 [1.46] NR | |
| ^a^ | Businelle et al. [44] | 2014 | NC | - | SMO. | 57 | M | NR | |
| ^a^ | Clasen et al. [45] | 2015 | NC | - | AFF. | 81 | Com | 28.72 [8.15] 19-55 | |
|  | Chandra et al. [34] | 2011 | NC | - | SMO. | 351 | Com | 39.5 [9.5] 21-65 | |
| ^a^ | Courvoisier et al. [46] | 2010 | NC | - | AFF. | 307 | NR | 22.79 [5.66] 14-64 | |
| ^a^ | Doherty et al. [47] | 2014 | NC | - | PHY. | 15 | Com | NR [NR] 20-45 | |
|  | Dunbar et al. [92] | 2010 | NC | - | SMO. | 394 | Com | 39.26 [9.55] NR | |
| ^a^ | Fitzsimmons-Craft et al. [48] | 2016 | NC | - | COG. | 232 | EI | 18.70 [1.00] 17-22 | |
| ^a^ | Fonareva et al. [49] | 2012 | NC | - | AFF. | 41 | M | 66.4 [7.9] NR | |
| ^a^ | Heron et al. [50] | 2014 | NC | - | EAT. | 127 | EI | 19.6 [1.18] NR | |
| ^a^ | Heron et al. [51] | 2013 | NC | - | PER. | 63 | EI | 19.04 [0.79] 18-22 | |
| ^a^ | Hofmann et al. [52] | 2014 | NC | - | PER. | 1,252 | Com | NR [NR] 18-68 | |
|  | Hofmann et al. [93] | 2014 | NC | - | EAT. | 204 | EI | 25.24 [6.32] 18-55 | |
|  | Hughes et al. [94] | 2015 | NC | - | AFF. | 120 | EI | 19.72 [1.58] 18-25 | |
|  | Huguet et al. [95] | 2015 | NC | - | SYM. | 65 | Com | NR [NR] 14-28 | |
| ^a^ | Johnson et al. [21] | 2009 | NC | - | AFF. | 280 | EI | 19.4 [1.4] NR | |
| ^a^ | Juth et al. [53] | 2015 | NC | - | AFF. | 117 | EI | 20 [2.07] 18-36 | |
|  | Kanning et al. [96] | 2016 | NC | - | PHY. | 69 | Com | 60.1 [7.1] 50-70 | |
| ^a^ | Kashdan et al. [54] | 2010 | NC | - | AFF. | 38 | Com | 26.9 [5.6] 21-50 | |
|  | Kashdan et al. [97] | 2014 | NC | - | AFF. | 42 | Com | 28.5 [8.6] NR | |
|  | Kimhy et al. [22] | 2014 | NC | - | AFF. | 27 | Com | 23.95 [5.01] NR | |
| ^a^ | Kirchner et al. [55] | 2013 | NC | - | SMO. | 475 | Com | 44.71 [11.05] NR | |
| ^a^ | Komulainen et al. [56] | 2014 | NC | - | PER. | 104 | EI | 23 [3.69] 19-35 | |
|  | Kuntsche et al. [38] | 2013 | NC | - | ALC. | 175 | EI | 23.4 [4.2] NR | |
|  | Kwapil et al. [98] | 2011 | NC | - | PER. | 305 | EI | 19.6 [2.8] NR | |
| ^a^ | Lange et al. [57] | 2014 | NC | - | COG. | 91 | Com | 67.97 [4.04] 60-76 | |
| ^a^ | Liao et al. [58] | 2015 | NC | - | PHY. | 114 | Com | NR [NR] 27–73 | |
|  | Luczak et al. [112] | 2015 | NC | - | ALC. | 32 | EI | 23.1 [2.71] NR | |
|  | MacKerron et al. [24] | 2013 | NC | - | AFF. | 21,947 | Com | NR | |
|  | Monk et al. [113] | 2015 | NC | - | ALC. | 69 | EI | 21.47 [4.47] 18 -36 | |
|  | Ottaviani et al. [37] | 2015 | NC | - | PER. | 42 | EI | 26.7 [7.7] NR | |
| ^a^ | Ramirez et al. [59] | 2014 | NC | - | ALC. | 42 | Com | 18.5 [1.2] 15–20 | |
| ^a^ | Riedinger et al. [60] | 2011 | NC | - | AFF. | 378 | Com | 42.5 [19.0] 14.0-86.5 | |
| ^a^ | Ritz et al. [23] | 2010 | NC | - | PHY. | 20 | Com | 31.6 [5.9] NR | |
| ^a^ | Robertson et al. [61] | 2012 | NC | - | ALC. | 404 | EI | 23.5 [7.6] NR | |
| ^a^ | Rowan et al. [62] | 2007 | NC | - | SMO. | 64 | Com | 41.8 [10.7] NR | |
| ^a^ | Rutledge et al. [63] | 2009 | NC | - | AFF. | 304 | W | Nurse=39.3 [9.8] NR; Physio=30.2 [4.5] NR | |
| ^a^ | Schuster et al. [64] | 2016 | NC | - | SMO. | 287 | EI | 21.3 [0.8] NR | |
|  | Schüz et al. [99] | 2015 | NC | - | EAT. | 53 | Com | 28.17 [11.15] 18-60 | |
|  | Schüz et al. [100] | 2014 | NC | - | SMO. | 119 | S | 42.64 [13.82] NR | |
|  | Schwerdtfeger et al. [101] | 2010 | NC | - | PHY. | 124 | Com | 31.67 [12.56] 18-73 | |
| ^a^ | Seto et al. [65] | 2016 | NC | - | EAT. | 12 | EI | 24.6 [3.06] 18-31 | |
| ^a^ | Setodji et al. [66] | 2014 | NC | - | SMO. | 128 | EI | NR [NR] 18-24 | |
| ^a^ | Shiffman et al. [67] | 2007 | NC | - | SMO. | 214 | S | 42 [10.2] NR | |
| ^a^ | Simons et al. [68] | 2016 | NC | - | ALC. | 263 | EI | 19.88 [1.37] 18-27 | |
| ^a^ | Spook et al. [69] | 2013 | NC | - | EAT. | 30 | EI | NR [NR] 16-21 | |
| ^a^ | Thielsch et al. [70] | 2015 | NC | - | PER. | 41 | EI | 23.08 [2.77] 19-32 | |
| ^a^ | Thomas et al. [36] | 2011 | NC | - | EAT. | 39 | EI | 20.1 [2.0] NR | |
|  | Tomiyama et al. [111] | 2009 | NC | - | EAT. | 127 | EI | 19.4 [2.18] 17-33 | |
| ^a^ | Tiplady et al. [71] | 2009 | NC | - | ALC. | 38 | Com | 22.8 [NR] 18-54 | |
|  | Trampe et al. [25] | 2015 | NC | - | AFF. | 11572 | Com | 32.9 [10.4] NR | |
|  | Warthen et al. [102] | 2009 | NC | - | SMO. | 43 | Com | 39.6 [10.9] 20-67 | |
| ^a^ | Waters et al. [72] | 2014 | NC | - | SMO. | 62 | S | 43.3 [11.1] NR | |
| ^a^ | Witkiewitz et al. [73] | 2012 | NC | - | SMO. | 86 | EI | 20.1 [1.7] NR | |
|  | Wray et al. [28] | 2016 | NC | - | MIS. | 12 | Com | 30 [8.8] 22-50 | |
| ^a^ | Zenk et al. [74] | 2014 | NC | - | EAT. | 100 | EI | 44.3 [10.5] NR | |
| ^a^ | Zawadzki et al. [29] | 2015 | NC | - | MIS. | 115 | Com | 41.2 [11.6] 19-63 | |
| ^a^ | Aaron et al. [75] | 2006 | C | TMJ | SYM. | 137 | M | 38 [11] NR | |
|  | Ainsworth et al. [110] | 2013 | C | Schizophrenia | SYM. | 24 | M | 33.0 [9.5] 18-49 | |
| ^a^ | Cook et al. [31] | 2010 | C | HIV | MIS. | 17 | M | NR | |
| ^a^ | Dewey et al. [76] | 2015 | C | PTSD | SYM. | 32 | EI | 20.3 [3.53] NR | |
| ^a^ | Dhingra et al. [77] | 2014 | C | Chronic pain | SMO. | 36 | M | 49.5 [10.3] NR | |
|  | eSa et al. [103] | 2016 | C | Psychosis | AFF. | 23 | Com | 26 [19-51] NR | |
|  | Ebner-Priemer et al. [104] | 2008 | C | Methadone | AFF. | 50 | M | 31.3 [8.1] NR | |
| ^a^ | Epstein et al. [78] | 2014 | C | Methadone | AFF. | 27 | M | 41.2 [7.7] 21–55 | |
| ^a^ | Fitzsimmons-Craft et al. [79] | 2015 | C | Anorexia | EAT. | 118 | Com+M | 25.3 [8.4] 18-58 | |
| ^a^ | Granholm et al. [27] | 2013 | C | Schizophrenia/  schizoaffective disorder | MIS. | 145 | Com+S | 46.5 [11.2] NR | |
|  | Green et al. [105] | 2016 | C | PTSD | SYM. | 83 | NR | 30.19 [5.44] NR | |
| ^a^ | Hachizuka et al. [80] | 2010 | C | Cancer (Hospice) | SYM. | 15 | C | 67.1 [9.2] 55-91 | |
| ^a^ | Hacker et al. [33] | 2007 | C | Cancer | SYM. | 20 | M | 48.7 [NR] 23-64 | |
| ^a^ | Johnson et al. [21] | 2009 | C | Schizophrenia | AFF. | 47 | S+M | 44.1 [10.5] NR | |
| ^a^ | Johnson et al. [21] | 2009 | C | Substance use | AFF. | 80 | S+M | 35.1 [9.6] NR | |
| ^a^ | Johnson et al. [21] | 2009 | C | Anxiety | AFF. | 39 | S+M | 29.1 [8.5] NR | |
| ^a^ | Jean et al. [26] | 2013 | C | Stroke | MIS. | 36 | M | 61.31 [12.94] NR | |
| ^a^ | Juengst et al. [81] | 2015 | C | TBI | AFF. | 17 | Com+M | 36.7 [12.4] NR | |
|  | Kaplan et al. [32] | 2016 | C | Oral | MIS. | 22 | C | 39.1 [12.2] NR | |
|  | Kimhy et al. [22] | 2014 | C | Schizophrenia | AFF. | 77 | M | 32.51 [9.19] NR | |
|  | Kothari et al. [107] | 2015 | C | Fibromyalgia | SYM. | 220 | Com+S | 51.25 [11.02] NR | |
| ^a^ | Kulich et al. [82] | 2015 | C | COPD | SYM. | 209 | S | NR [NR] 44-86 | |
| ^a^ | Kuroi et al. [83] | 2015 | C | Oral | SYM. | 25 | M | 59.3 [7.92] NR | |
| ^a^ | Lavender et al. [84] | 2013 | C | Anorexia | AFF. | 118 | Com+M | 25.3 [8.4] 18-58 | |
|  | Mazure et al. [106] | 2014 | C | Stroke | AFF. | 43 | M | 59.5 [12.8] NR | |
| ^a^ | Merwin et al. [85] | 2015 | C | T1DM | AFF. | 74 | C | 41.89 [12.43] NR | |
| ^a^ | Munsch et al. [86] | 2009 | C | Binge ED | EAT. | 21 | C | 45.1 [11.1] NR | |
| ^a^ | Okifuji et al. [87] | 2011 | C | Fibromyalgia | SYM. | 81 | Com+S | 28.75 [6.24] NR | |
| ^a^ | Ritz et al. [23] | 2010 | C | Asthma | PHY. | 20 | M | 28.0 [6.8] NR | |
|  | Santangelo et al. [114] | 2014 | C | BPD/PTSD/Bulimia | AFF. | 119 | Com+M | 28.71 [8.07] NR | |
|  | Smyth et al. [109] | 2007 | C | Bulimia Nervosa | AFF. | 131 | S | 25.3 [7.6] NR | |
| ^a^ | Sohl et al. [88] | 2008 | C | Chronic Fatigue | SYM. | 53 | S | 41.7 [9.6] NR | |
|  | Solhan et al. [19] | 2009 | C | BPD | AFF. | 58 | Com | 32.3 [11.2] NR | |
|  | Solhan et al. [19] | 2009 | C | MDD/Dysthymia | AFF. | 42 | Com | 35.4 [11.7] NR | |
| ^a^ | Sorbi et al. [20] | 2006 | C | Chronic pain (3-6 mo) | SYM. | 15 | Com+S | 40.6 [6.7] NR | |
| ^a^ | Sorbi et al. [20] | 2006 | C | Chronic pain (6-12 mo) | SYM. | 25 | Com+S | 40.6 [6.7] NR | |
| ^a^ | Sorbi et al. [20] | 2006 | C | Chronic pain (>12 mo) | SYM. | 40 | Com+S | 40.6 [6.7] NR | |
| ^a^ | Thielsch et al. [89] | 2015 | C | GAD | AFF. | 56 | S | 38.25 [13.47] NR | |
|  | Thomas et al. [36] | 2011 | C | Abdominal surgery | EAT. | 21 | M | 48.5 [12.6] NR | |
| ^a^ | Weaver et al. [90] | 2007 | C | Cancer | SYM. | 6 | M | 64 [NR] 54-76 | |
|  | Yoshiuchi et al. [35] | 2007 | C | Chronic Fatigue | SYM. | 9 | NR | NR | |
| ^a^ Study included in meta-analysis; AFF, affect; C, clinical; Com, community; ED, eating disorder; BPD, borderline personality disorder; COG, cognitions; COPD, chronic obstructive pulmonary disease; EAT, eating; ED, electronic diary; EI, educational institution; GAD, generalised anxiety disorder; M, medical centre; MDD, major depressive disorder; MIS, miscellaneous; NC, nonclinical; NR, not reported; PER, personality; PHY, physical activity; PTSD, post-traumatic stress disorder; S, sample from larger study; SMO, smoking; SYM, symptoms; T2DM, type 2 diabetes; TBI, traumatic brain injury; TMJ, Temporomandibular Disorder | | | | | | | | |  |

|  | | | | | | | | | | | | | | | | |
| --- | --- | --- | --- | --- | --- | --- | --- | --- | --- | --- | --- | --- | --- | --- | --- | --- |
| **Table 2** Ecological momentary assessment (EMA) protocol and compliance characteristics for studies included within review (ordered by first author surname in nonclinical and clinical populations). | | | | | | | | | | | | | | | | |
| **Study** | | | **EMA protocol** | | | | | | | | | | **Compliance** | | | |
| *^a^ = Included in MA* | |  | Device | Training: Yes (1); No (0) | Incentives | Incentive thresholds: Yes (1); No (0) | Monitoring duration (days) | Prompt/d (time-based) (max) | Prompts/participant study duration | Items/time-based prompt (min.) | # prompt types | BURDEN SCORE | Attrition (%) | Compliance (%) | Compliance after data  exclusions yes (1) no (0) | |
| ^a^ | Ambwani et al. [39] | 2015 | PALMT | 1 | CC | 1 | 14 | 6 | 84 | 15 | 1 | 1260 | NR | 95.60 | 0 | |
| ^a^ | Andersson et al. [40] | 2007 | MOB | 0 | F | 1 | 7 | 3 | 21 | 25 | 2 | 1050 | NR | 97.00 | 0 | |
| ^a^ | Armey et al. [30] | 2011 | PALMT | 1 | CC/F | 0 | 7 | 6 | 42 | 28 | 2 | 2352 | NR | 38.00 | 0 | |
|  | Asselbergs et al. [91] | 2016 | MOB | 0 | F | 1 | 42 | 5 | 210 | 3 | 1 | 630 | 79 | NR | 0 | |
| ^a^ | Berg et al. [41] | 2014 | PALMT | 1 | F | 1 | 14 | 6 | 84 | 17 | 3 | 4284 | 100 | 90.00 | 0 | |
| ^a^ | Buckner et al. [42] | 2012 | PALMT | 1 | CC | NR | 14 | 7 | 98 | 6 | 3 | 1764 | 82 | 62.50 | 0 | |
|  | Burns et al. [108] | 2015 | PALMT | 0 | F | 0 | 14 | 5 | 70 | 4 | 1 | 280 | 87 | NR | 0 | |
| ^a^ | Burt et al. [43] | 2010 | PALMT | 1 | CC | NR | 7 | 6 | 42 | 32 | 1 | 1344 | 100 | 54.90 | 0 | |
| ^a^ | Businelle et al. [44] | 2014 | MOB | 1 | F | 1 | 6 | 4 | 24 | 26 | 1 | 624 | 84 | 82.70 | 0 | |
| ^a^ | Clasen et al. [45] | 2015 | MOB | 1 | F | 1 | 21 | 5 | 105 | 3 | 1 | 315 | 87 | 77.68 | 0 | |
|  | Chandra et al. [34] | 2011 | PALMT | 1 | N | NA | 16 | 5 | 80 | 7 | 2 | 1120 | 85 | NR | 0 | |
| ^a^ | Courvoisier et al. [46] | 2010 | MOB | 0 | F | 0 | 7 | 6 | 42 | 4 | 1 | 168 | 96 | 74.90 | 0 | |
| ^a^ | Doherty et al. [47] | 2014 | MOB | 1 | F | 0 | 1 | 13 | 13 | 8 | 2 | 208 | NR | 75.00 | 0 | |
|  | Dunbar et al. [92] | 2010 | PALMT | 1 | F | 0 | 12 | 5 | 60 | 22 | 1 | 1320 | NR | NR | 0 | |
| ^a^ | Fitzsimmons-Craft et al. [48] | 2016 | MOB | 1 | CC | NR | 14 | 3 | 42 | 13 | 2 | 1092 | 99 | 89.30 | 0 | |
| ^a^ | Fonareva et al. [49] | 2012 | PALMT | 1 | N | NA | 1 | 4 | 4 | 5 | 1 | 20 | NR | 85.00 | 0 | |
| ^a^ | Heron et al. [50] | 2014 | PALMT | 1 | F | 0 | 7 | 5 | 35 | 9 | 1 | 315 | 97 | 90.00 | 0 | |
| ^a^ | Heron et al. [51] | 2013 | PALMT | 1 | CC | NR | 7 | 5 | 35 | 10 | 1 | 350 | NR | 75.00 | 0 | |
| ^a^ | Hofmann et al. [52] | 2014 | MOB | 0 | N | NA | 3 | 5 | 15 | 14 | 1 | 210 | NR | 70.50 | 0 | |
|  | Hofmann et al. [93] | 2014 | MOB | 1 | F | 1 | 7 | 7 | 49 | 11 | 1 | 539 | 98 | NR | 0 | |
|  | Hughes et al. [94] | 2015 | PALMT | 1 | CC/F | 0 | 7 | 4 | 28 | 10 | 1 | 280 | 79 | NR | 0 | |
|  | Huguet et al. [95] | 2015 | MOB | 0 | F | 0 | 14 | 1 | 14 | 16 | 2 | 448 | NR | NR | 0 | |
| ^a^ | Johnson et al. [21] | 2009 | PALMT | 1 | F | 0 | 7 | 5 | 35 | NR | 1 | UTD | 71 | 83 | 0 | |
| ^a^ | Juth et al. [53] | 2015 | PALMT | 1 | F | 0 | 5 | 6 | 30 | 27 | 1 | 810 | 94 | 73.00 | 0 | |
|  | Kanning et al. [96] | 2016 | MOB | 1 | F | 0 | 3 | NR | UTD | 6 | 1 | UTD | 92 | NR | 0 | |
| ^a^ | Kashdan et al. [54] | 2010 | PALMT | 1 | F | 1 | 14 | 4 | 56 | 28 | 1 | 1568 | NR | 81.00 | 1 | |
|  | Kashdan et al. [97] | 2014 | PALMT | 1 | F | 1 | 14 | 5 | 70 | 16 | 2 | 2240 | NR | NR | 0 | |
|  | Kimhy et al. [22] | 2014 | PALMT | 0 | NR | NR | 2 | 10 | 20 | 4 | 1 | 80 | NR | NR | 0 | |
| ^a^ | Kirchner et al. [55] | 2013 | MOB | 0 | N | NA | 27 | 3 | 81 | 2 | 1 | 162 | 98 | 79.00 | 0 | |
| ^a^ | Komulainen et al. [56] | 2014 | PALMT | 0 | N | NA | 7 | 10 | 70 | 14 | 1 | 980 | 98 | 76.90 | 0 | |
|  | Kuntsche et al. [38] | 2013 | MOB | 0 | O | 1 | 30 | 3 | 90 | 11 | 1 | 990 | 90 | NR | 0 | |
|  | Kwapil et al. [98] | 2011 | PALMT | 0 | N | NA | 7 | 8 | 56 | 24 | 1 | 1344 | 94 | NR | 0 | |
| ^a^ | Lange et al. [57] | 2014 | MOB | 1 | N | NA | 7 | 4 | 28 | 13 | 1 | 364 | NR | 77.00 | 0 | |
| ^a^ | Liao et al. [58] | 2015 | MOB | 0 | F | 0 | 4 | 8 | 32 | 14 | 1 | 448 | NR | 82.00 | 0 | |
|  | Luczak et al. [112] | 2015 | MOB | 1 | F | 0 | 14 | NR | UTD | 14 | 3 | UTD | NR | 91.00 | 0 | |
|  | MacKerron et al. [24] | 2013 | MOB | 0 | N | NA | UNL | 2 | UTD | 6 | 1 | UTD | NR | NR | 0 | |
|  | Monk et al. [113] | 2015 | MOB | 0 | N | NA | 7 | NR | UTD | 5 | 2 | UTD | NR | 69.00 | 1 | |
|  | Ottaviani et al. [37] | 2015 | MOB | 1 | F | 0 | 1 | 36 | 36 | 17 | 2 | 1224 | 94 | NR | 0 | |
| ^a^ | Ramirez et al. [59] | 2014 | MOB | 1 | N | NA | 7 | 4 | 28 | 2 | 3 | 168 | NR | 85.60 | 0 | |
| ^a^ | Riedinger et al. [60] | 2011 | MOB | 1 | F | 0 | 9 | 6 | 54 | 18 | 1 | 972 | NR | 89.7 | 0 | |
| ^a^ | Ritz et al. [23] | 2010 | OTHER | 1 | F | 0 | 21 | 3 | 63 | 4 | 2 | 504 | NR | 94.90 | 0 | |
| ^a^ | Robertson et al. [61] | 2012 | PALMT | 1 | F | 0 | 21 | 1 | 21 | 1 | 1 | 21 | NR | 89.00 | 0 | |
| ^a^ | Rowan et al. [62] | 2007 | PALMT | 1 | F | 1 | 7 | 4 | 28 | 8 | 3 | 672 | 66 | 69.20 | 0 | |
| ^a^ | Rutledge et al. [63] | 2009 | PALMT | 1 | N | NA | 7 | 6 | 42 | 19 | 2 | 1596 | NR | 73.30 | 1 | |
| ^a^ | Schuster et al. [64] | 2016 | PALMT | 1 | F | 1 | 7 | 7 | 49 | 13 | 2 | 1274 | 100 | 92.60 | 0 | |
|  | Schüz et al. [99] | 2015 | MOB | 1 | F | 0 | 10 | 5 | 50 | 19 | 3 | 2850 | NR | NR | 0 | |
|  | Schüz et al. [100] | 2014 | MOB | 1 | F | 1 | 10 | 5 | 50 | NR | 3 | UTD | 95 | NR | 0 | |
|  | Schwerdtfeger et al. [101] | 2010 | PALMT | 1 | CC | NR | 1 | 12 | 12 | 14 | 2 | 336 | NR | NR | 0 | |
| ^a^ | Seto et al. [65] | 2016 | MOB | 1 | N | NA | 14 | 5 | 70 | 6 | 2 | 840 | NR | 50 | 1 | |
| ^a^ | Setodji et al. [66] | 2014 | PALMT | 1 | F | 1 | 21 | 3 | 63 | 7 | 2 | 882 | 96 | 83.00 | 0 | |
| ^a^ | Shiffman et al. [67] | 2007 | PALMT | 1 | N | NA | 28 | 5 | 140 | 8 | 2 | 2240 | NR | 91.00 | 1 | |
| ^a^ | Simons et al. [68] | 2016 | PALMT | 1 | F | 1 | 49 | 7 | 343 | 10 | 1 | 3430 | 96 | 79 | 1 | |
| ^a^ | Spook et al. [69] | 2013 | MOB | 1 | F | 0 | 7 | 5 | 35 | NR | 1 | UTD | NR | 40.00 | 0 | |
| ^a^ | Thielsch et al. [70] | 2015 | iPod | 1 | N | NA | 7 | 7 | 49 | 2 | 1 | 98 | NR | 90.40 | 0 | |
| ^a^ | Thomas et al. [36] | 2011 | PALMT | 1 | F | 1 | 10 | 6 | 60 | 4 | 1 | 240 | 91 | 71 | 1 | |
|  | Tomiyama et al. [111] | 2009 | PALMT | 1 | N | NA | 2 | UTD^b^ | UTD | 14 | 1 | UTD | 97 | 67 | 1 | |
| ^a^ | Tiplady et al. [71] | 2009 | MOB | 1 | N | NA | 14 | 2 | 28 | NR | 1 | UTD | 95 | 80.3 | 1 | |
|  | Trampe et al. [25] | 2015 | MOB | 0 | N | NA | 8.6^c^ | 6 | 52 | 1 | 1 | 52 | NR | NR | 0 | |
|  | Warthen et al. [102] | 2009 | PALMT | 1 | F | 1 | 8 | 4 | 32 | 9 | 1 | 288 | NR | NR | 0 | |
| ^a^ | Waters et al. [72] | 2014 | PALMT | 1 | F | 1 | 7 | 4 | 28 | 1 | 3 | 84 | 92 | 74 | 1 | |
| ^a^ | Witkiewitz et al. [73] | 2012 | MOB | 1 | F | 1 | 21 | 3 | 63 | 8 | 2 | 1008 | NR | 86.20 | 0 | |
|  | Wray et al. [28] | 2016 | MOB | 1 | F | 1 | 30 | 8 | 240 | 5 | 3 | 3600 | NR | NR | 0 | |
| ^a^ | Zenk et al. [74] | 2014 | MOB | 1 | F | 0 | 7 | 5 | 35 | 22 | 1 | 770 | 98 | 68.90 | 0 | |
| ^a^ | Zawadzki et al. [29] | 2015 | PALMT | 1 | F | 1 | 3 | 6 | 18 | 10 | 1 | 180 | NR | 84.00 | 0 | |
| ^a^ | Aaron et al. [75] | 2006 | PALMT | 1 | F | 1 | 14 | 3 | 42 | 35 | 1 | 1470 | 79 | 90 | 1 | |
|  | Ainsworth et al. [110] | 2013 | MOB | 1 | F | NR | 12 | 4 | 48 | 18 | 1 | 864 | 100 | UTD | 0 | |
| ^a^ | Cook et al. [31] | 2010 | PALMT | 1 | F | 1 | 180 | 1 | 180 | 26 | 1 | 4680 | 67 | 75.8 | 0 | |
| ^a^ | Dewey et al. [76] | 2015 | MOB | 1 | CC | NR | 14 | 6 | 84 | 22 | 1 | 1848 | NR | 67.50 | 0 | |
| ^a^ | Dhingra et al. [77] | 2014 | PALMT | 1 | NR | NR | 7 | 6 | 42 | 7 | 2 | 588 | 100 | 68.00 | 0 | |
|  | eSa et al. [103] | 2016 | PALMT | 1 | NR | NR | 6 | 10 | 60 | 17 | 1 | 1020 | NR | NR | 0 | |
|  | Ebner-Priemer et al. [104] | 2008 | PALMT | 1 | F | NR | 1 | NR | UTD | 9 | 1 | UTD | NR | NR | 0 | |
| ^a^ | Epstein et al. [78] | 2014 | PALMT | NR | NR | NR | 112 | 3 | 336 | 27 | 2 | 18144 | 100 | 79.00 | 1 | |
| ^a^ | Fitzsimmons-Craft et al. [79] | 2015 | PALMT | 1 | F | 1 | 14 | 7 | 98 | 54 | 3 | 15876 | 97 | 87 | 1 | |
| ^a^ | Granholm et al. [27] | 2013 | PALMT | 1 | F | NR | 7 | 4 | 28 | NR | 1 | UTD | 62 | 72.1 | 0 | |
|  | Green et al. [105] | 2016 | PALMT | NR | NR | NR | 1 | NR | UTD | NR | 1 | UTD | 97 | NR | 0 | |
| ^a^ | Hachizuka et al. [80] | 2010 | PALMT | 1 | NR | NR | 7 | 5 | 35 | 6 | 3 | 630 | 97 | 90.3 | 0 | |
| ^a^ | Hacker et al. [33] | 2007 | WATCH | NR | NR | NR | 6 | 3 | 18 | 1 | 1 | 18 | 97 | 87 | 0 | |
| ^a^ | Johnson et al. [21] | 2009 | PALMT | 1 | F | NR | 7 | 4 | 28 | NR | 1 | UTD | 91 | 69 | 0 | |
| ^a^ | Johnson et al. [21] | 2009 | PALMT | 1 | F | NR | 7 | 4 | 28 | NR | 1 | UTD | 95 | 80 | 0 | |
| ^a^ | Johnson et al. [21] | 2009 | PALMT | 1 | F | NR | 7 | 4 | 28 | NR | 1 | UTD | 94 | 73 | 0 | |
| ^a^ | Jean et al. [26] | 2013 | PALMT | 1 | NR | NR | 7 | 5 | 35 | 3 | 1 | 105 | 88 | 74.13 | 0 | |
| ^a^ | Juengst et al. [81] | 2015 | MOB | 1 | NR | NR | 42 | 3 | 126 | 5 | 1 | 630 | 96 | 73.4 | 0 | |
|  | Kaplan et al. [32] | 2016 | PALMT | 1 | F | 1 | 7 | 8 | 56 | 11 | 1 | 616 | 99 | NR | 0 | |
|  | Kimhy et al. [22] | 2014 | PALMT | NR | NR | NR | 2 | 10 | 20 | 4 | 1 | 80 | NR | NR | 0 | |
|  | Kothari et al. [107] | 2015 | MOB | 1 | F | 1 | 21 | 4 | 84 | 4 | 1 | 336 | NR | UTD | 0 | |
| ^a^ | Kulich et al. [82] | 2015 | ED | NR | NR | NR | 182 | 0 | 364 | 7 | 1 | 2548 | NR | 86.00 | 1 | |
| ^a^ | Kuroi et al. [83] | 2015 | MOB | 1 | NR | NR | 7 | UTD^b^ | 12 | 2 | 1 | UTD | NR | 90.6 | 0 | |
| ^a^ | Lavender et al. [84] | 2013 | PALMT | 1 | F | 1 | 14 | 7 | 98 | 2 | 3 | 588 | 97 | 87.00 | 1 | |
|  | Mazure et al. [106] | 2014 | PALMT | 1 | NR | NR | 7 | 5 | 35 | 10 | 1 | 350 | 98 | NR | 0 | |
| ^a^ | Merwin et al. [85] | 2015 | MOB | 1 | NR | NR | 3 | 42 | 126 | 6 | 2 | 1512 | 91 | 96.46 | 0 | |
| ^a^ | Munsch et al. [86] | 2009 | PALMT | 1 | NR | NR | 7 | 5 | 35 | NR | 2 | UTD | 93 | 68.00 | 1 | |
| ^a^ | Okifuji et al. [87] | 2011 | PALMT | NR | NR | NR | 30 | 3 | 90 | 8 | 1 | 720 | 100 | 80.20 | 0 | |
| ^a^ | Ritz et al. [23] | 2010 | OTHER | 1 | F | NR | 21 | 3 | 63 | 4 | 2 | 504 | NR | 97.00 | 1 | |
|  | Santangelo et al. [114] | 2014 | PALMT | 1 | F | NR | 1 | UTD^b^ | UTD | 5 | 1 | UTD | NR | 94 | 0 | |
|  | Smyth et al. [109] | 2007 | PALMT | 1 | F | 1 | 14 | 7 | 98 | 73 | 3 | 21462 | 88 | NR | 0 | |
| ^a^ | Sohl et al. [88] | 2008 | PALMT | 1 | F | NR | 21 | 6 | 126 | 2 | 1 | 252 | NR | 93.00 | 0 | |
|  | Solhan et al. [19] | 2009 | PALMT | 1 | F | 1 | 28 | 6 | 168 | 31 | 1 | 5208 | NR | NR | 0 | |
|  | Solhan et al. [19] | 2009 | PALMT | 1 | F | 1 | 28 | 6 | 168 | 31 | 1 | 5208 | NR | NR | 0 | |
| ^a^ | Sorbi et al. [20] | 2006 | PALMT | 1 | NR | NR | 28 | 4 | 112 | 31 | 1 | 3472 | NR | 86 | 1 | |
| ^a^ | Sorbi et al. [20] | 2006 | PALMT | 1 | NR | NR | 28 | 4 | 112 | 31 | 1 | 3472 | NR | 86 | 1 | |
| ^a^ | Sorbi et al. [20] | 2006 | PALMT | 1 | NR | NR | 28 | 4 | 112 | 31 | 1 | 3472 | NR | 93 | 1 | |
| ^a^ | Thielsch et al. [89] | 2015 | iPod | 1 | NR | NR | 7 | 4 | 28 | 1 | 2 | 56 | NR | 86.00 | 0 | |
|  | Thomas et al. [36] | 2011 | PALMT | 1 | F | 1 | 6 | 2 | 12 | 2 | 2 | 48 | NR | UTD | UTD | |
| ^a^ | Weaver et al. [90] | 2007 | MOB | 1 | NR | NR | 42 | 2 | 84 | 6 | 1 | 504 | NR | 98.00 | 0 |  |
|  | Yoshiuchi et al. [35] | 2007 | WATCH | 1 | NR | NR | 21 | UTD^b^ | UTD | 18 | 2 | UTD | NR | NR | 0 |  |
| ^a^ Study included in meta-analysis; ^b^ prompts/d unable to be determined as prompt schedule based on wake hours, which were not specified; ^c^ median; CC, course credit; ED, electronic diary; EI, educational institution; F, financial; MOB, mobile; NR, not reported; PALMT, palmtop computer; S, sample from larger study; UNL, unlimited; UTD, unable to be determined | | | | | | | | | | | | | | | |  |
