# Supplementary material for: Compliance With Mobile Ecological Momentary Assessment of Self-Reported Health-Related Behaviors and Psychological Constructs in Adults: Systematic Review and Meta-analysis
Source: J Med Internet Res. 2021 Mar 3;23(3):e17023. doi: 10.2196/17023 (PMC7970161; doi:10.2196/17023)
Supplement: Multimedia Appendix 2 [file jmir_v23i3e17023_app2.docx]

**Multimedia Appendix 2**

Summary of mobile ecological momentary assessment (mEMA) targets and participant characteristics in nonclinical and clinical mEMA studies.

| **Primary mEMA^a^ target** | Data sets: NC^b^ or C^c^, (n=) | Secondary mEMA targets^d^ | | | | | | | | | Sample included in analysis | | Recruitment source^e^ | | | | | |
| --- | --- | --- | --- | --- | --- | --- | --- | --- | --- | --- | --- | --- | --- | --- | --- | --- | --- | --- |
|  |  | Smoking | Alcohol | Eating | Physical activity | Affect | Cognitions | Symptoms | Physical environment | Social environment | Sample size total; median (range) | Median of mean age (range), years | Educational institution | Community | Workplace | Health service | Prior study | NR^f^ |
| **Smoking** | NC (12) | —^g^ | 1  (8) | — | 3  (25) | 10  (83) | 4  (33) | — | 4  (33) | 4  (33) | 2280; 124 (43-475) | 41  (20-45) | 3  (25) | 5  (42) | — | 1  (8) | 3  (25) | — |
|  | C^h^ (1) | — | — | — | — | — | — | 1  (100) | — | — | 36 (NA^i^) | 50 (NA) | — | — | — | 1  (100) | **—** | — |
| **Alcohol** | NC (8) | — | — | — | — | 7  (88) | 1  (13) | 1  (13) | 5  (63) | 4  (50) | 1038; 56 (15-404) | 23 (19-27) | 6 (75) | 2 (25) | — | — | — | — |
|  | C (0) | — | — | — | — | — | — | — | — | — | — | — | — | — | — | — | — | — |
| **Eating behaviors** | NC (10) | — | — | — | 3  (30) | 9  (90) | — | — | 3  (30) | 4  (40) | 782; 52  (12-204) | 25 (19-44) | 8  (80) | 2  (20) | — | — | — | — |
|  | C (3) | — | — | — | 1  (33) | 1  (33) | 1  (33) | 1  (33) | — | 1  (33) | 160; 21  (21-118) | 45  (25-49) | — | 2  (67) | — | 2  (67) | — | — |
| **Physical activity** | NC (5) | — | — | — | — | 3  (60) | 1  (20) | — | 2  (40) | 4  (80) | 342; 69 (15-124) | 32  (32-60) | — | 5  (10) | — | — | — | — |
|  | C (1) | — | — | — | — | — | — | 1  (100) | — | 1  (100) | 20; (NA) | 28 (NA) | — | — | — | 1  (100) | — | — |
| **Other** | NC (3) | — | 1  (33) | — | 1  (33) | 2  (66) | — | — | — | 1  (33) | 163; 36 (12-115) | 30  (19-41) | 1  (33) | 2  (66) | — | — | — | — |
|  | C (4) | — | — | — | 1  (25) | 2  (50) | 2  (50) | — | 1  (25) | 2  (50) | 220; 29 (17-145) | 47  (39-61) | — | 2  (50) | — | 2  (50) | — | 1  (25) |
| **Personality traits** | NC (7) | 1  (14) | 1  (14) | 1  (14) | 4  (57) | 7  (100) | 4  (57) | — | 1  (14) | 2  (29) | 1910; 103 (41-1252) | 21  (19-27) | 6  (86) | 1  (14) | — | — | — | — |
|  | C (0) | — | — | — | — | — | — | — | — | — | — | — | — | — | — | — | — | — |
| **Affect** | NC (15) | 2  (13) | — | 3  (20) | 5  (33) | — | 4  (27) | 2  (13) | 2  (13) | 4  (27) | 35330; 117 (27-21947) | 25  (19-66) | 5  (33) | 7  (47) | 1  (7) | 1  (7) | — | 1  (7) |
|  | C (16) | — | 3  (19) | 3  (19) | 3  (19) | — | 3  (19) | 6  (38) | 2  (13) | 6  (38) | 1001; 53 (17-131) | 34  (25-60) | — | 7  (44) | — | 10  (63) | 5  (31) | — |
| **Cognitions** | NC (2) | — | — | 1  (50) | 1  (50) | 1  (50) | — | — | — | — | 323; 162 (91-232) | 43  (19-68) | 1  (50) | 1  (50) | — | — | — | — |
|  | C (0) | — | — | — | — | — | — | — | — | — | — | — | — | — | — | — | — | — |
| **Symptom** | NC (2) | — | — | — | — | 2  (10) | — | — | — | — | 170; 85 (65-105) | 46 (NR) | — | 1  (50) | — | 1  (50) | — | — |
|  | C (16) | — | — | — | 3  (19) | 6  (38) | 4  (25) | — | — | — | 994; 29  (6-220) | 41  (20-67) | 1  (6) | 6  (38) | — | 5  (31) | 7  (44) | 2  (13) |
| **Total** | NC (64) | 3  (5) | 3  (5) | 5  (8) | 17  (27) | 41  (64) | 14  (22) | 3  (5) | 17  (27) | 23  (36) | 42338; 89 (12-21947) | 25  (19-68) | 3  (5) | 26  (41) | 1  (2) | 3  (5) | 3  (5) | 1  (2 |
|  | C (41) | — | 3  (7) | 3  (7) | 8  (20) | 9  (22) | 10  (24) | 9  (22) | 3  (7) | 10  (24) | 2431; 40 (6-220) | 39  (20-67) | 1  (2) | 17  (41) | 0  (0) | 21  (51) | 12  (30) | 3  (7) |
|  | T^j^ (105) | 3  (2.8) | 6  (5.7) | 8  (7.6) | 25  (23.8) | 50  (47.6) | 24  (22.8) | 12  (11.4) | 20  (19.0) | 33  (31.4) | 44769; 62 (6-21947) | 31  (19-68) | 3  (2.8) | 43  (40.9) | 1  (0.9) | 24  (22.8) | 15  (14.2) | 4  (3.8) |

^a^mEMA: mobile ecological momentary assessment.

^b^NC: nonclinical.

^c^C: clinical.

^d^Studies could include more than 1 mEMA target.

^e^Studies could recruit from more than 1 source

^f^NR: not reported.

^g^Blank cells indicate data set did not collect mEMA data for this target behavior or psychological construct

^h^C: clinical.

^i^NA: not available as domain includes a single study

^j^T: total with nonclinical and clinical data sets pooled.
