# Supplementary material for: Compliance With Mobile Ecological Momentary Assessment of Self-Reported Health-Related Behaviors and Psychological Constructs in Adults: Systematic Review and Meta-analysis
Source: J Med Internet Res. 2021 Mar 3;23(3):e17023. doi: 10.2196/17023 (PMC7970161; doi:10.2196/17023)
Supplement: Multimedia Appendix 3 [file jmir_v23i3e17023_app3.docx]

**Multimedia Appendix 3**

Summary of mobile ecological momentary assessment protocols.

| Primary mEMA^a^ target | NC^b^  or C^c^,  n= | Device,  n (%) | | | Monitoring duration (days),  median (range) | Training provided n (%) | Incentive provided n (%) | Notification types, n (%) | | | Prompts/d, median (range) | Items /prompt, median (range) | Response latency | |
| --- | --- | --- | --- | --- | --- | --- | --- | --- | --- | --- | --- | --- | --- | --- |
|  |  | Palmtop/PDA | Mobile phone | Other |  |  |  | Random (signal) | Interval (fixed) | Event-based |  |  | Criteria reported n (%) | Criteria (min), median (range) |
| **Smoking** | NC (12) | 8 (67) | 4  (33) | —^d^ | 11  (6-28) | 11 (92) | 9  (75) | 4 (33) | 5 (42) | 3 (25) | 4  (3-7) | 8  (1-26) | 4  (33) | 3  (2-4) |
|  | C (1) | 1  (100) | — | — | 7  (NA^e^) | 1 (100) | 0 | — | 1 (100) | — | 6 (NA) | 7 (NA) | 1 (100) | 15  (NA) |
| **Alcohol** | NC (8) | 2  (25) | 6  (75) | — | 14  (7-49) | 5  (63) | 5  (63) | 4 (50) | 2 (25) | 2 (25) | 3  (1-7) | 10  (1-25) | — | — |
|  | C (0) | — | — | — | — | — | — | — | — | — | — | — | — | — |
| **Eating behaviors** | NC (10) | 5  (50) | 5  (50) | — | 9  (2-14) | 10 (100) | 8  (80) | 7 (70) | 1 (10) | 2 (20) | 5  (5-7) | 14  (4-22) | 2  (20) | 38  (30-45) |
|  | C (3) | 3 (100) | — | — | 7  (6-14) | 3 (100) | 2 (67) | — | 2  (67) | 1 (33) | 5  (2-7) | 28  (2-54) | 2  (67) | 38  (30-45) |
| **Physical activity** | NC (5) | 1  (20) | 3  (60) | 1 (20) | 3  (1-21) | 4 (80) | 5 (100) | 2 (40) | 3  (60) | — | 10  (3-13) | 8  (4-14) | 2  (40) | 8  (5-10) |
|  | C (1) | — | — | 1 (100) | 21 (NA) | 1 (100) | 1 (100) | 0  (0) | 1 (100) | — | 3  (NA) | 4  (NA) | — | — |
| **Other** | NC (3) | 2  (66) | 1  (33) |  | 7  (3-30) | 3 (100) | 3 (100) | 1 (33) | 1 (33) | 1 (33) | 6  (6-8) | 10  (5 -28) | 1  (33) | 2  (2-2) |
|  | C (4) | 4 (100) | — |  | 7  (7-180) | 4 (100) | 3  (75) | 4  (100) | — | — | 5  (1-8) | 11  (3-26) | 3  (75) | 15  (1.5-30) |
| **Personality traits** | NC (7) | 4  (57) | 2  (29) | 1 (14) | 7  (1-7) | 4 (57) | 3  (43) | 6 (86) | 1 (14) | — | 7  (5-36) | 14  (2-42) | 2  (29) | 15  (10-20) |
|  | C (0) | — | — | — | — | — | — | — | — | — | — | — | — | — |
| **Affect** | NC (15) | 9  (60) | 6  (40) | — | 8  (1-42) | 10 (67) | 10 (67) | 12 (80) | 2 (13) | 1 (7) | 5  (2-10) | 6  (1-28) | 5  (33) | 15  (2-60) |
|  | C (16) | 13 (81) | 2  (13) | 1  (6) | 7  (1-112) | 14 (88) | 9  (56) | 11 (73) | 3 (20) | 2 (13) | 6  (3-42) | 9  (1-73) | 9  (56) | 15  (15-60) |
| **Cognitions** | NC (2) | — | 2 (100) | — | 11  (7-14) | 2 (100) | 1  (50) | 1 (50) | 1 (50) | — | 4  (3-4) | 13  (13-13) | — | — |
|  | C (0) | — | — | — | — | — | — | — | — | — | — | — | — | — |
| **Symptoms** | NC (2) | 1 (50) | 1  (50) | — | 14  (14-14) | 0  (0) | 2 (100) | 1 (50) | 1 (50) | — | 3  (1-5) | 10  (4-16) | — | — |
|  | C (16) | 8  (50) | 5  (31) | 3 (19) | 21  (1-182) | 12 (75) | 5  (31) | 14 (88) | 1  (6) | 1 (6) | 4  (2-6) | 8  (1-35) | 7  (44) | 10  (5-45) |
| **Total** | NC (64) | 32 (50) | 30 (47) | 2  (3) | 7  (1-49) | 49 (76) | 46 (72) | 38 (59) | 17 (27) | 9 (14) | 5  (1-36) | 10  (1-32) | 16 (25) | 10  (2-60) |
|  | C (41) | 29 (71) | 7 (17) | 5 (12) | 12  (1-182) | 35 (85) | 20 (49) | 29 (71) | 8 (20) | 4 (10) | 4 (1-42) | 8  (1-73) | 22 (54) | 15  (1.5-60) |
|  | T^f^ (105) | 61 | 37 | 7 | 7  (1-182) | 84 | 66 | 67 | 25 | 13 | 5  (1-42) | 10  (1-73) | 38 | 15  (1.5-60) |
|  | T (%) | 58.0 | 35.2 | 6.6 | — | 80.0 | 62.8 | 63.8 | 23.8 | 12.3 | — | — | 36.1 | — |

^a^mEMA: mobile ecological momentary assessment.

^b^NC: nonclinical.

^c^C: clinical.

^d^Blank cells indicate data not applicable

^e^NA: not available as domain includes a single study.

^f^T: total with nonclinical and clinical data sets pooled.
